# NG2 Proteoglycan Enhances Brain Tumor Progression by Promoting Beta-1 Integrin Activation in both Cis and Trans Orientations

**DOI:** 10.3390/cancers9040031

**Published:** 2017-03-31

**Authors:** William B. Stallcup

**Affiliations:** Sanford Burnham Prebys Medical Discovery Institute, Cancer Center, Tumor Microenvironment and Cancer Immunology Program, 10901 North Torrey Pines Road, La Jolla, CA 92037, USA; stallcup@SBPdiscovery.org; Tel.: +1-858-646-3100

**Keywords:** brain tumor progression, brain tumor vascularization, NG2 proteoglycan, beta-1 integrin, pericyte, endothelial cell, macrophage, tumor vessel function, Cre-Lox gene ablation

## Abstract

By physically interacting with beta-1 integrins, the NG2 proteoglycan enhances activation of the integrin heterodimers. In glioma cells, co-localization of NG2 and α3β1 integrin in individual cells (cis interaction) can be demonstrated by immunolabeling, and the NG2-integrin interaction can be confirmed by co-immunoprecipitation. NG2-dependent integrin activation is detected via use of conformationally sensitive monoclonal antibodies that reveal the activated state of the beta-1 subunit in NG2-positive versus NG2-negative cells. NG2-dependent activation of beta-1 integrins triggers downstream activation of FAK and PI3K/Akt signaling, resulting in increased glioma cell proliferation, motility, and survival. Similar NG2-dependent cis activation of beta-1 integrins occurs in microvascular pericytes, leading to enhanced proliferation and motility of these vascular cells. Surprisingly, pericyte NG2 is also able to promote beta-1 integrin activation in closely apposed endothelial cells (trans interaction). Enhanced beta-1 signaling in endothelial cells promotes endothelial maturation by inducing the formation of endothelial junctions, resulting in increased barrier function of the endothelium and increased basal lamina assembly. NG2-dependent beta-1 integrin signaling is therefore important for tumor progression by virtue of its affects not only on the tumor cells themselves, but also on the maturation and function of tumor blood vessels.

## 1. Introduction

**Figure 1:**
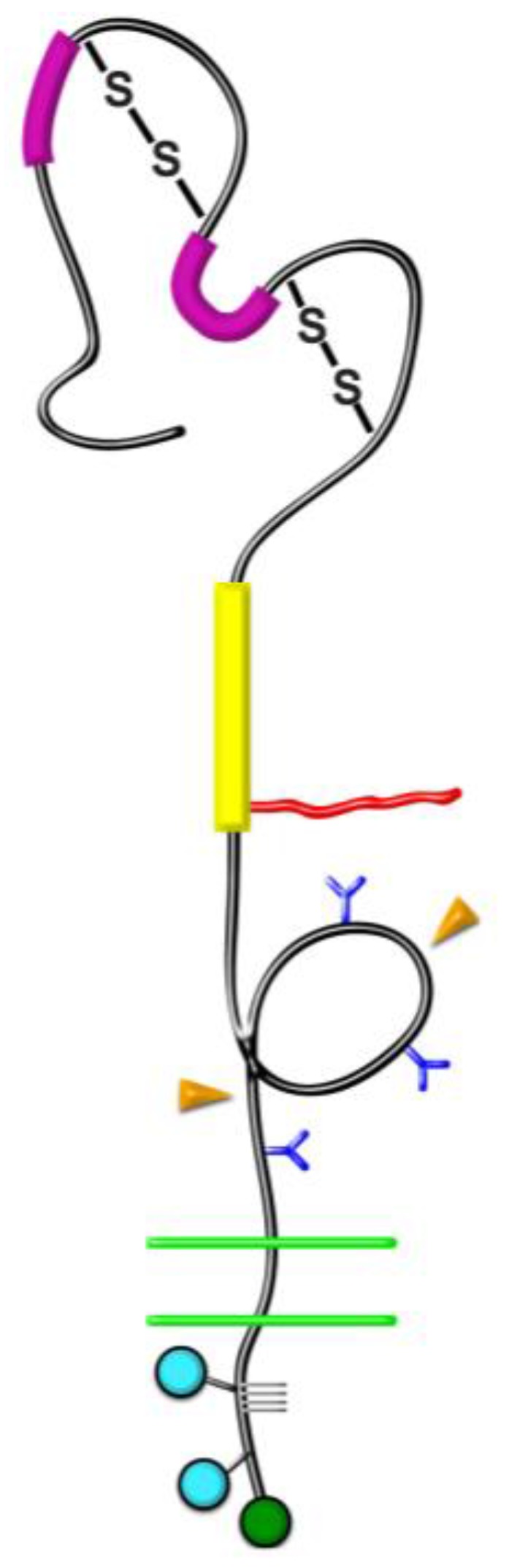
Domain structure of NG2. NG2 is a type-1 transmembrane protein with several distinct structural domains. *Domain 1*. Bold magenta bars = laminin G domains; S-S = disulfide bonds. *Domain 2.* Bold yellow bar = collagen binding domain; Irregular red line = chondroitin sulfate chain attached at S-999. *Domain 3*. Blue Y shapes = N-linked oligosaccharides; Orange arrowheads = sites of proteolytic cleavage. *Transmembrane domain*. Double green lines = plasma membrane. *Cytoplasmic domain*. Blue circles = sites of threonine phosphorylation at T-2256 and T-2314; Green circle = C-terminal PDZ-binding motif; Gray grid lines = proline-rich segment. Figure taken from [17].

The membrane-spanning NG2 proteoglycan (Figure 1), also known as chondroitin sulfate proteoglycan-4 (CSPG4), has only limited signal transducing capability of its own [1,2]. However, the ability of NG2 to promote signaling through β1 integrins [3,4,5,6,7,8,9] and through receptor tyrosine kinases [10,11,12,13,14,15] makes the proteoglycan a significant accessory contributor to transmembrane signaling. Studies with a number of different immature or progenitor cell types indicate that NG2-enhanced signaling processes promote cell proliferation, motility, and survival, all of which are valuable properties for progenitor cells [16,17]. These characteristics would also be expected to benefit tumor progression, and indeed, expression of NG2 is correlated with tumor malignancy and poor patient outcome in several types of cancer [3,18,19,20,21,22,23,24,25,26]. NG2 knockdown impairs tumor progression [3], and in the one instance where it has been tested, forced expression of NG2 in melanoma cells has been shown to enhance tumor growth [27]. In this respect, most research has been focused on NG2 expression in the case of gliomas and melanomas, since NG2 is often found on the tumor cells themselves in these neoplasms. However, because NG2 is also expressed by several elements of the tumor stroma, including macrophages and microvascular pericytes [28], NG2 expression is also relevant to many other cancers in which the tumor cells are NG2-negative. Although the properties of tumor cells are obviously important in dictating malignancy, the impact of host stromal elements is proving to be surprisingly powerful in determining the aggressiveness of tumor growth and spread. Therefore, in order to appreciate the full impact of NG2 on tumor progression, it is important to understand mechanisms by which the proteoglycan exerts it effects not only on tumor cells, but also on various types of host cells. In this review, we will focus on the NG2-dependent activation of β1 integrins as a mechanism that impacts the contributions of both tumor and stromal cells to tumor progression. A key aspect of this research has been the finding that NG2 is capable of activating β1 integrins when the two molecules are expressed in the same cell (cis interaction), as well as when the two molecules are expressed by two different cells that are closely apposed (trans interaction).

## 2. Cis Interaction of NG2 with β1 Integrins

When expressed in the same cell, NG2 and β1 integrins co-localize and interact physically, promoting an activated conformation for the integrin that has important consequences for cell behavior. These cis interactions occur in both tumor and stromal cells.

### 2.1. NG2 in Glioma Cells

**Figure 2:**
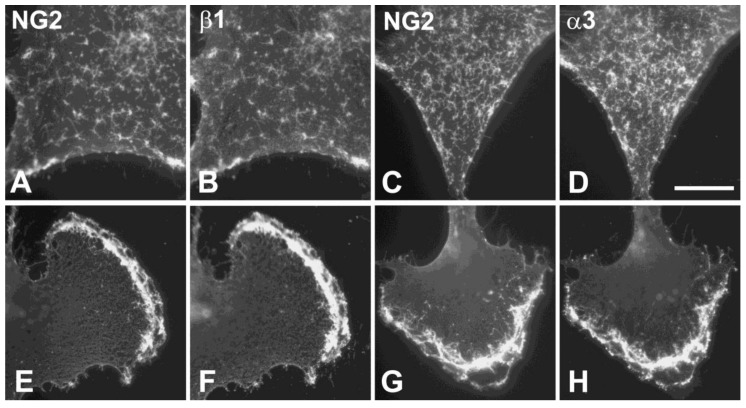
NG2 and α3β1 integrin co-localization on U251 glioma cells. NG2-transfected U251 cells were double immunolabeled with rabbit anti-NG2 (**A**,**C**,**E**,**G**) and mouse monoclonal antibody against either β1 (**B**,**F**) or α3 (**D**,**H**) integrin. (**A**–**D**) U251 cells expressing the T2314E NG2 variant. NG2 and α3β1 co-localize to microprotrusions from the apical cell surface. (**E**–**H**) U251 cells expressing the T2256E NG2 variant. NG2 and α3β1 co-localize to prominent lamellipodia on the leading cell edge. Bar in (**D**) = 5 μm.

Using detergent extracts of the NG2-expressing U87 human glioma cell line, we have demonstrated a physical interaction between NG2 and α3β1 integrin by showing that the integrin co-immunoprecipitates with NG2. Similar results were obtained with the NG2-negative human U251 glioma cell line following expression of full-length rat NG2 [3]. Although glycosaminoglycan chains are often important for proteoglycan function [29,30], we have not detected a role for the single NG2 chondroitin sulfate chain in NG2 interaction with integrins, suggesting that the interaction is protein-protein in nature. We also used U251 cells to investigate changes in cell biology that are caused by forced expression of several key NG2 variants [7,8]. These single amino acid variants are relevant to the phosphorylation status of the NG2 cytoplasmic domain, which can be modified at Thr-2256 by protein kinase C-alpha (PKCα) and at Thr-2314 by the proline-dependent kinase ERK. The T2256E NG2 variant mimics the PKCα-phosphorylated state of NG2, while the T2314E variant mimics the ERK-phosphorylated state. Conversely, the T2256V and T2314V variants represent non-phosphorylatable forms of NG2. When expressed in U251 cells, all of these forms of NG2 co-localize with α3β1 integrin, consistent with our evidence that the interaction between the two molecules is mediated by the NG2 ectodomain rather than the cytoplasmic domain. However, there are two distinct sites of NG2-α3β1 co-localization, which are dictated by the phosphorylation state of the proteoglycan (Figure 2). When phosphorylated at Thr-2314, NG2 and its α3β1 binding partner localize to arrays of microprotrusions on the apical cell surface of U251 cells (Figure 2A–D). The phosphomimetic T2314E NG2 variant is also highly concentrated in these microprotrusions, while the non-phosphorylatable T2314V variant fails to occupy these arrays. Electron microscopy of immune-gold labeled cells reveals these NG2-rich structures to resemble microvilli averaging about 2 μm in length [7]. Very similar NG2-rich microprotrusions are seen on the apical surfaces of melanoma cells [31]. In contrast, when phosphorylated at Thr-2256, NG2 and its associated α3β1 partner localize to broad lamellipodia (Figure 2E–H). The phosphomimetic T2256E variant also spontaneously localizes to lamellipodia, while the T2256V variant fails to do so [8]. In wound healing (scratch) assays performed in vitro, it is apparent that these NG2-rich lamellipodia represent the leading edges of motile cells [7].

**Figure 3:**
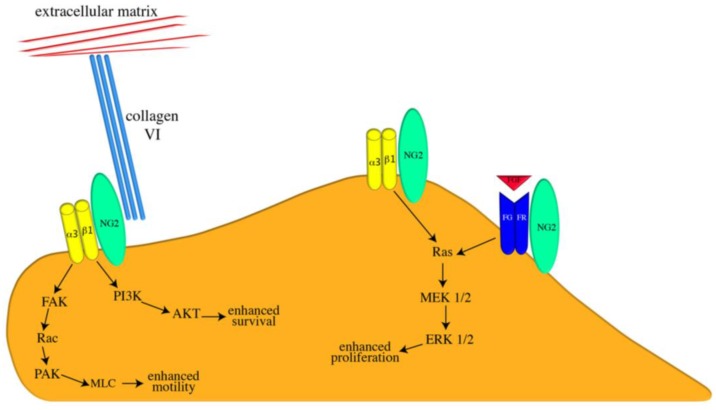
Spatial localization of functional NG2 interactions. On the apical cell surface, the T-2314 phosphorylated NG2 proteoglycan (green oval) activates α3β1 integrin (yellow dimer) signaling to promote enhanced proliferation. NG2 also promotes cell proliferation via potentiation of growth factor/growth factor receptor signaling (indicated here by red FGF and blue FGFR dimer). On leading edge lamellipodia, T-2256 phosphorylated NG2 activates α3β1 integrin signaling to promote enhanced motility. NG2-dependent integrin signaling can also enhance cell survival via the PI3K/AKT pathway. In addition, NG2 provides a linkage between the cell surface and the extracellular matrix via its interaction with type VI collagen (triple turquoise rods). Signaling pathways are shown in abbreviated format due to space consideration. Figure taken from [17].

We hypothesize that the site of NG2 phosphorylation determines the ability of its cytoplasmic domain to interact with known intracellular scaffolding partners such as ezrin, MUPP1, GRIP1, and syntenin-1 [7,8,32,33,34]. In turn, these cytoplasmic interactions dictate the localization of the NG2-α3β1 complex to specific membrane microdomains in apical microprotrusions or lamellipodia. The functional importance of these localization patterns becomes apparent when we compare the cell biology of the NG2 variants. While motility cannot be stimulated in T2256V variants, the T2256E species is spontaneously motile, with NG2-positive lamellipodia forming the leading edges of migrating cells. Increased motility is also seen in wild type NG2 transfectants in which PKCα activity is stimulated by treatment with phorbol, 12-myristate, 13-acetate (PMA). In contrast, T2314E variants exhibit spontaneously increased proliferation, a property also seen in wild type NG2 transfectants in which ERK is directly stimulated by the upstream presence of constitutively active MEK-DD. Predictably, proliferation cannot be stimulated in T2314V variants. The importance of α3β1 in mediating either motility or proliferation is indicated by the ability of anti-β1 antibody to inhibit both processes. In addition, immunolabeling with the conformationally-sensitive HUTS-21 monoclonal antibody [35] reveals that the β1 integrin subunit is highly activated in association with NG2 in both T2256E lamellipodia and in TT2314E microprotrusions [8]. Interestingly, motility and proliferation appear to be mutually exclusive. Spontaneously motile T2256E variants are very poorly proliferative, while spontaneously proliferative T2314E variants exhibit very low motility. We propose that the phosphorylation state of NG2, determined by the balance of PKCα and ERK activities that are controlled by the cellular environment, is an important aspect of the cell’s choice to proliferate or migrate. As a result of its phosphorylation, the anchorage pattern of NG2 is able to direct α3β1 localization to specific membrane microdomains where it can interact with machinery that promotes either motility (lamellipodia) or proliferation (microprotrusions). A representation of these localized signaling mechanisms is shown in Figure 3. 

**Figure 4:**
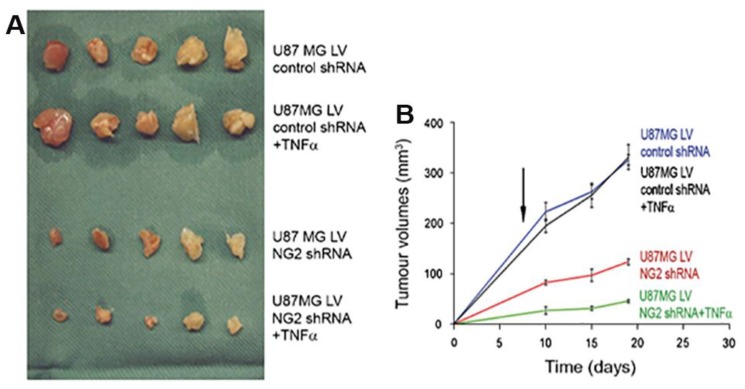
Effect of NG2 knockdown on growth of U87MG gliomas. Cohorts of 5 NOD-SCID mice were innoculated subcutaneously with 5 × 10^6^ U87MG glioma cells that had been transduced with lentivirus (LV) for expression of either control shRNA or NG2 shRNA. NG2 expression was reduced by 75% in the NG2 shRNA group compared to the control shRNA group. One cohort of mice from each tumor group also received IP injections of TNFα (150 μg/kg) once daily on days 3–10 following tumor cell injections. A. Final tumor sizes in the 4 cohorts of mice. B. Kinetics of tumor growth in the 4 cohorts of mice. NG2 knockdown significantly slows U87MG tumor growth, likely due to loss of NG2-dependent cell proliferation. The NG2 knockdown tumors exhibit an additional sensitivity to treatment with TNFα (lacking in the control tumors) due to loss of NG2 pro-survival effects. Data taken from [3].

Another important consequence of NG2-dependent β1 integrin activation is an increase in PI3K/AKT survival signaling [3]. Use of the conformationally-sensitive HUTS-21 antibody reveals that β1 integrin activation occurs in NG2-expressing U87 glioma cells in similar fashion to that observed in NG2-transfected U251 cells. In both glioma cell lines, β1 activation is accompanied by increased phosphorylation of AKT. This NG2-dependent survival signaling via the PI3/AKT axis results in increased glioma cell resistance to treatment with chemotherapeutic drugs, including carboplatin, cisplatin, doxorubicin, and TNFα. This drug resistance is absent in NG2-negative parental U251 cells and in NG2-knockdown U87 cells. The involvement of PI3K/AKT signaling is indicated by increased sensitivity to drug treatment in the presence of the PI3K inhibitor wortmannin. The importance of upstream β1 integrin activation in drug resistance is demonstrated by the ability of anti-β1 antibodies to increase drug sensitivity. The relevance of these findings to glioma growth in vivo is emphasized by the reduced growth of NG2-knockdown U87 tumors compared to control NG2-positive U87 tumors, likely due to diminished cell proliferation in the knockdown tumors. In addition, the effect of NG2 expression on drug resistance is demonstrated by an even further reduction in growth of the NG2-knockdown tumors as a result of administration of TNFα [3]. In contrast, growth of control, NG2-positive U87 tumors is unaffected by TNFα administration (Figure 4).

An intriguing, but untested, possibility related to glioma cell survival is suggested by the finding that expression of α6β1 integrin by glioma stem cells is important for interaction of these cells with laminin in the perivascular niche [36]. This niche enhances survival and helps maintain stemness in this population. A cis interaction of NG2 with α6β1 on the glioma stem cell surface might be important for promoting activation of the integrin to enhance its binding to laminin in the perivascular niche.

### 2.2. NG2 in Pericytes

Vascularization is a key aspect of tumor progression, with the vascular switch serving as the point at which tumors have recruited sufficient vasculature to expand beyond a very small volume [37,38]. The interaction between endothelial cells and smooth muscle-like pericytes is critical for microvessel formation, maturation, maintenance, and remodeling [39,40]. Our ability to identify and study pericytes at an early point in their participation in microvessel development has been much improved by using NG2 as a marker for immature pericytes [41,42,43]. In addition, our work with germline NG2 null mice [12] has indicated that NG2-deficient pericytes are restricted in their ability to support neovascularization. This is emphasized in retinal and corneal models of neovascularization, which reveal reduced recruitment and participation of NG2 null pericytes in the formation of new blood vessels [10,44].

The importance of NG2-dependent β1 integrin activation in pericyte recruitment has been demonstrated more clearly by in vitro studies using human brain microvascular pericytes [9]. siRNA-mediated knockdown of NG2 in pericytes leads to a 60% decrease in β1 integrin activation (judged by immunolabeling with the conformationally-sensitive HUTS-21 antibody), further resulting in a 40% reduction in phosphorylation of the downstream effector FAK. This decrease in NG2-dependent β1/FAK signaling has an important impact on pericyte behavior. NG2 knockdown pericytes exhibit a 4-fold decrease in proliferation and a 3-fold decrease in PDGF-BB stimulated migration. These changes in pericyte biology are likely to underlie the deficits in recruitment of NG2 null pericytes seen in retinal and corneal angiogenesis models [10,44]. 

### 2.3. NG2 in Macrophages

**Figure 5:**
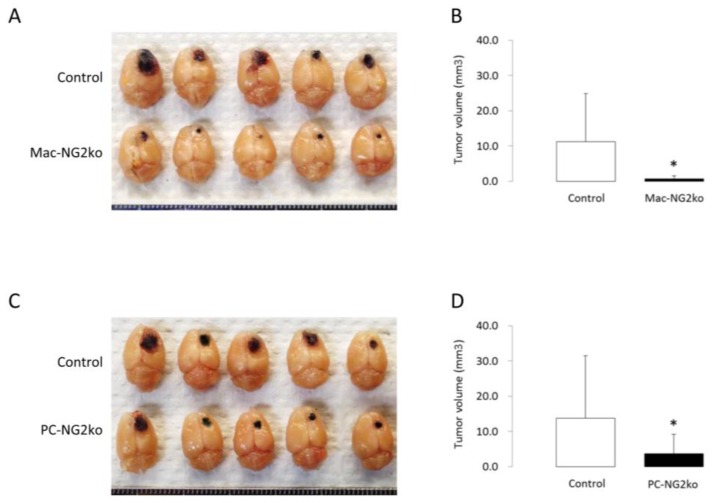
Decreased brain tumor progression in myeloid and pericyte-specific NG2 null mice. Although primary melanomas arise mostly in the skin, the brain is a common site for melanoma metastasis [56]. We used intracranial B16F10 melanomas as a model of melanoma growth in the brain. B16F10 tumors in control versus Mac-NG2ko mice (A) and in control versus PC-NG2ko mice (C) were evaluated at 10 days after tumor initiation via intracranial microinjection (2 × 10^4^ cells per mouse). Graphs quantify tumor volumes in control versus Mac-NG2ko mice (B) and in control versus PC-NG2ko mice (D). Data in B represent 21 control and 20 Mac-NG2ko mice. Data in D represent 22 control and 18 PC-NG2ko mice. *p < 0.01 compared to controls. Data taken from [48].

Preliminary data suggest that cis interactions between NG2 and integrins may also be important for macrophage recruitment to tumors. We generated myeloid-specific NG2 null (Mac-NG2ko) mice by crossing NG2 floxed mice [45] with LysM-Cre mice [46,47]. Growth of intracranial B16F10 melanomas is greatly retarded in Mac-NG2ko mice compared to tumor growth in control mice (Figure 5A,B), a phenomenon that correlates with significantly reduced accumulation of macrophages in tumors in the NG2 null mice [28,48]. In the absence of large numbers of tumor macrophages, vascularization of tumors in Mac-NG2ko mice is greatly impaired, possibly due to loss of macrophage-derived factors that stimulate tumor vascularization [49,50]. In vitro studies with human THP1 macrophages reveal that NG2 knockdown results in loss of activation of both β1 and β2 integrins in these cells [28], as judged by immunolabeling with the conformationally sensitive HUTS-21 and mAb-24 [51] antibodies, respectively. We hypothesize that NG2-dependent activation of macrophage α4β1 and αMβ2 may be important for binding of these integrins to VCAM1 and ICAM1 expressed by endothelial cells [52,53,54,55]. These interactions are key aspects of myeloid cell arrest and subsequent transmigration across the vascular endothelium. Ablation of NG2 might weaken these interactions to an extent that impairs macrophage extravasation from the circulation into tumors. Confirmation of these possibilities requires additional experimentation.

## 3. Trans Interactions of NG2 with β1 Integrins

In addition to its ability to activate β1 integrins in a cis orientation, NG2 also appears able to activate β1 in a trans orientation when the two molecules are expressed on two different closely apposed cells. While this was initially surprising to us, this phenomenon has a precedent in the case of homotypic interactions between cadherin molecules, which can occur in both cis and trans orientations [57,58]. Interactions between Eph receptors and ephrins are also observed to occur in both cis and trans orientations [59]. The intimate interaction between pericytes and endothelial cells in microvessels provides a good example of trans interaction between NG2 and β1 integrin.

### 3.1. Effects of Soluble NG2 on Endothelial Cells

An understudied aspect of NG2 biology is the proteolytic shedding of the proteoglycan’s ectodomain from cell surfaces [60,61,62,63] (see also Figure 1). While neither the precise mechanism underlying NG2 shedding [64] nor the functional significance of shedding [65,66] are well-understood, it is significant that the shed ectodomain can substitute for membrane-bound NG2 in supporting certain signaling functions, including potentiation of growth factor signaling [10]. Extending this line of investigation, we found that soluble, recombinant NG2 ectodomain [67] is effective in promoting angiogenesis in a corneal vascularization assay [4]. This action of NG2 is based on its ability to stimulate endothelial cell motility, morphogenesis, and tube formation, as demonstrated using in vitro assays. We subsequently identified α3β1 integrin and galectin-3 as NG2 binding partners on the endothelial cell surface, and demonstrated the ability of soluble NG2 to activate β1 integrin in both mouse and human endothelial cells [4] via use of the conformationally sensitive β1 antibodies 9EG7 (mouse) [68] and HUTS-21 (human). These studies suggest the possibility that NG2 shed from pericytes might be able to act at a distance to aid in recruiting endothelial cells to sites of angiogenesis.

### 3.2. Effects of Pericyte NG2 on Endothelial Cells

**Figure 6:**
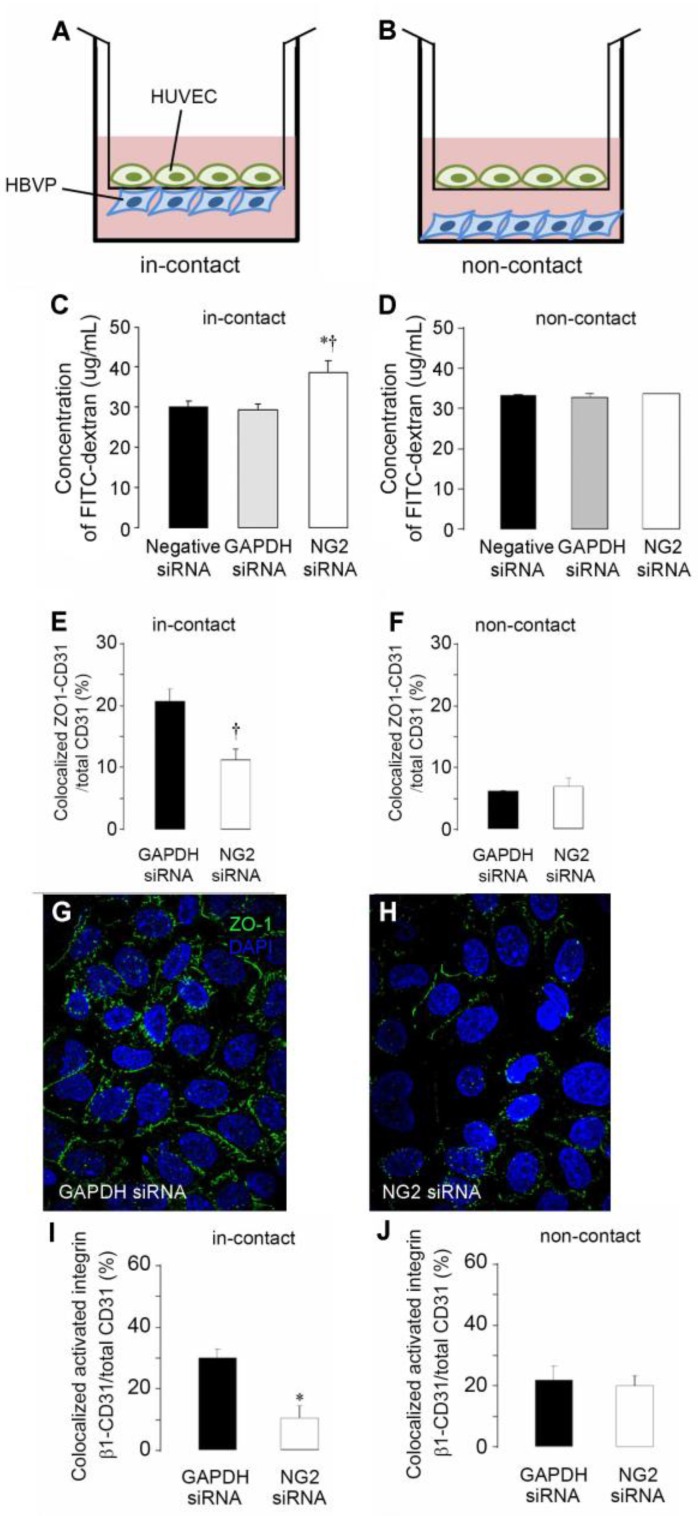
NG2 knockdown in pericytes reduces integrin activation and formation of endothelial junctions. Pericyte-endothelial cell interactions were investigated using in-contact (**A**) and non-contact (**B**) Transwell co-cultures. HUVEC: human umbilical vein endothelial cells. HBVP: human brain vascular pericytes. NG2 knockdown in pericytes reveals that NG2 expression is required for enhanced barrier function (reduced leakage of FITC-dextran from upper to lower chamber) by the endothelial monolayer in the in-contact model (**C**) but not in the non-contact model (**D**). GAPDH siRNA and negative (scrambled NG2) siRNA serve as controls for NG2 siRNA in these experiments. In the in-contact model (**E**), NG2 expression by pericytes is needed for formation of the ZO-1 positive endothelial junctions that underlie barrier function. Expression of NG2 by pericytes does not affect endothelial junction formation in the non-contact model (**F**). Junctions are quantified as percentage of CD31-positive cells with ZO-1 positive junctions. Robust formation of ZO-1 positive endothelial junctions (green) is observed when NG2-positive pericytes are used in the in-contact model (**G**: blue = DAPI). Endothelial junction formation is impaired in the in-contact model when NG2 is knocked down in pericytes (**H**). In the in-contact model, β1 integrin activation in endothelial cells is reduced by knockdown of NG2 in pericytes (**I**). In the non-contact model, endothelial β1 integrin activation is not affected by NG2 knockdown in pericytes (**J**). The HUTS-21 antibody was used to quantify β1 integrin activation in HUVECs. Activation is quantified as the percentage of CD31-positive cells with HUTS-21 positive junctions. Data taken from [9].

Our studies on the effects of soluble NG2 on endothelial cells further suggested the possibility that membrane-bound NG2 on pericyte surfaces could be involved in pericyte interaction with endothelial cells. This was tested in vitro using human pericyte and endothelial cells grown on opposite sides of transwell membranes with 0.4 μm pores [9]. These pores are too small to allow the passage of cells [69], but are large enough to allow contact between pericyte and endothelial cell processes, generating an “in-contact” model of pericyte-endothelial cell interaction (Figure 6A). A “non-contact” model is generated by growing endothelial cells on the membrane and pericytes on the bottom of the chamber Figure 6B). Compared to non-contact co-cultures, the presence of control, NG2-positive pericytes in the in-contact model improves the barrier function of the endothelial monolayer, as judged by decreased leakage of FITC-dextran from the upper to lower chamber Figure 6C,D). This decreased permeability is due to the formation of ZO-1 positive endothelial junctions in the endothelial monolayer under the influence of interactions with pericytes (Figure 6E–H). Increased endothelial junction formation and improved barrier function are not seen in the non-contact model or when NG2 knockdown pericytes are used in the assay, showing that cell-cell contact and NG2 expression are required for the effect of pericytes on endothelial cells. The effect of NG2 is based on NG2-dependent activation of β1 integrin signaling in the endothelial monolayer, as shown by immunolabeling with the conformationally-sensitive HUTS-21 antibody [9]. This ability to activate endothelial β1 integrin is lacking in NG2 knockdown pericytes (Figure 6I,J).

**Figure 7:**
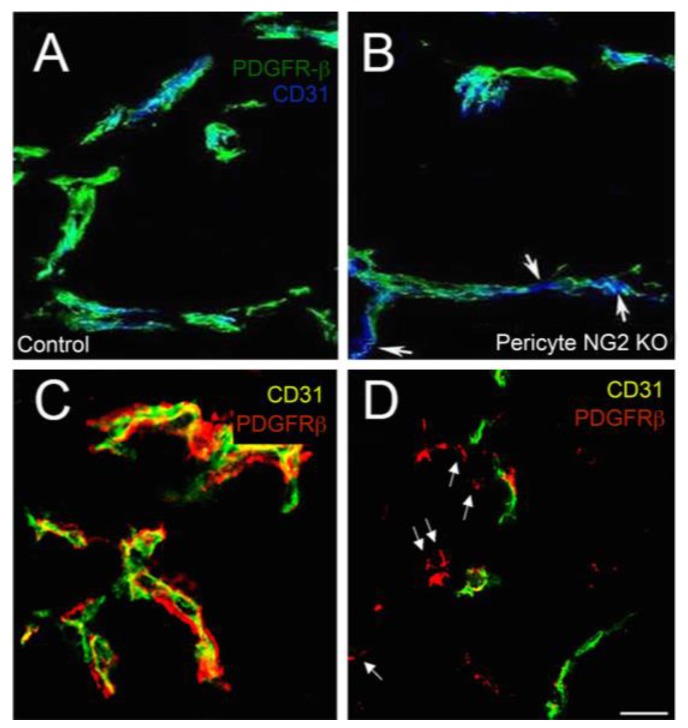
Pericyte ensheathment of endothelial cells in tumors in NG2 null mice. Vessels in intracranial B16F10 tumors were compared at 10 days in control (**A**) versus PC-NG2ko (**B**) mice, and in control (**C**) versus MacNG2ko (**D**) mice by immunolabeling sections for CD31 (endothelial cells) and PDGFRβ (pericytes). Pericyte (green) ensheathment of endothelial cells (blue) is incomplete in PC-NG2ko vessels (**B**), as judged by gaps in coverage (arrows). These gaps are not present in control vessels (**A**). In Mac-NG2ko vessels (**D**), many pericytes (red, arrows) fail to associate with endothelial cells (green). These detached pericytes are not seen in control vessels (**C**). These images also highlight the reduced diameter of vessels in Mac-NG2ko mice, a phenotype that is not observed in PC-NG2ko mice. Bar in D = 20 μm. Data taken from [9,48].

These in vitro findings provide insight into the deficits in vascularization of intracranial B16F10 tumors in pericyte-specific NG2 null mice (PC-NG2ko). These mice are generated by crossing NG2 floxed mice [45] with pdgfrb-Cre mice [70,71]. Greater than 95% of pericytes in PC-NG2ko mice are negative for NG2 [9,28,48]. Progression of intracranial melanomas is significantly impaired in PC-NG2ko mice, compared to control mice (Figure 5C,D). As initially suggested by work in the germline NG2 null mouse [72,73], this reduced tumor growth stems from deficits in the structure and function of tumor blood vessels [9,48]. Underlying these deficits in vascularization is a diminished ability of NG2 null pericytes to effectively ensheath endothelial cells, as judged by double immunolabeling for the endothelial marker CD31 and the pericyte marker PDGFRβ (Figure 7A,B). Pericyte coverage of the endothelial tube is reduced by almost 40% in tumors in PC-NG2ko mice. The decrease in this key interaction between pericytes and endothelial cells is accompanied by a 30% decrease in basal lamina assembly (as judged by collagen IV immunolabeling) and by a 35% decrease in endothelial junction formation (as revealed by ZO-1 immunolabeling), reminiscent of the in vitro results obtained with NG2 knockdown pericytes in transwell co-cultures with endothelial cells. As a result of these structural deficits in tumor blood vessels, vessel patency is decreased almost 2-fold, while vessel leakiness is increased almost 3-fold. A 5-fold increase in tumor hypoxia reflects the decreased blood flow to the tumors caused by these structural and functional deficiencies [9,28].

This change in pericyte-endothelial cell interaction in tumors in PC-NG2ko mice can be contrasted with the changes observed in tumors in Mac-NG2ko mice. Even though pericytes still express NG2 in the Mac-NG2ko mice, pericytes largely fail to interact with endothelial cells in tumors in these mice (Figure 7C,D). Structural and functional properties of tumor vessels in Mac-NG2ko mice are correspondingly diminished compared to PC-NG2ko mice [48], likely accounting for the slower tumor growth seen in myeloid-specific NG2 null mice (Figure 5A,B). These observations emphasize the fact that NG2 is only one of many functional players involved in determining the ability of pericytes to interact effectively with endothelial cells. 

## 4. Future Prospects

Our work clearly establishes the ability of the NG2 proteoglycan to activate β1 integrin signaling in the context of several processes that impact brain tumor progression. These include not only the malignant properties of the tumor cells, but also the properties of tumor blood vessels that are essential for tumor growth. Nevertheless, there are several aspects of the work in which additional mechanistic understanding is needed in order to fully appreciate the nature and importance of the NG2-integrin interaction.

1. What domains of NG2 and the integrin heterodimer are responsible for their interaction? Our results indicate that the NG2-integrin interaction is mediated by the extracellular domains of the two proteins, rather than by the cytoplasmic domains. However, we do not know what part of the NG2 ectodomain is involved in the interaction, or whether NG2 binds to the α or β integrin subunit (or to both). This also leaves open the questions of whether NG2 preferentially interacts with specific β1 heterodimers and how the spatial requirements for the interaction can be satisfied in both the cis and trans orientations.
2. Does NG2 influence crosstalk between integrins and receptor tyrosine kinases? Crosstalk between integrins and receptor tyrosine kinases is proposed to be an important aspect of amplifying and/or modulating transmembrane signaling [74,75,76,77]. Since NG2 has the ability to activate both of these signaling entities, it is of interest to know how the proteoglycan may affect crosstalk that occurs between the two and what implications this may have for the properties (e.g., proliferation, motility, survival) of both tumor and stromal cells.
3. How does NG2 interact with the cytoskeleton and how is this interaction affected by phosphorylation of the NG2 cytoplasmic domain? We propose that the phosphorylation state of NG2 determines its interaction with cytoplasmic scaffolding proteins and thus the localization of both NG2 and its β1 binding partner to specific membrane microdomains (lamellipodia versus apical microprotrusions). It remains for us to identify these cytoplasmic scaffolding proteins and to demonstrate how they anchor the NG2-integrin complex at sites that specifically influence proliferation versus motility.
4. How does NG2-dependent cis activation of integrins influence the biology of macrophages? Although we can demonstrate enhanced activation of both β1 and β2 integrins in NG2-positive THP1 macrophages, we have not yet established whether this influences macrophage interaction with the vascular endothelium (e.g. via binding to VCAM1 or ICAM1) or the ability of macrophages to extravasate from the circulation into tumors and other sites of inflammation. This will be an important addition to the spectrum of cellular events that are impacted by NG2.
